# The Mitochondrial-Derived Peptides, HumaninS14G and Small Humanin-like Peptide 2, Exhibit Chaperone-like Activity

**DOI:** 10.1038/s41598-017-08372-5

**Published:** 2017-08-10

**Authors:** Alan K. Okada, Kazuki Teranishi, Fleur Lobo, J. Mario Isas, Jialin Xiao, Kelvin Yen, Pinchas Cohen, Ralf Langen

**Affiliations:** 1 Department of Biochemistry and Molecular Biology, Zilkha Neurogenetic Institute, University of Southern California, Los Angeles, California 90033 USA; 2 University of Southern California Davis School of Gerontology, Ethel Percy Andrus Gerontology Center, University of Southern California, Los Angeles, CA 90089-0191 USA

## Abstract

Mitochondrial-derived peptides (MDPs) and their analogs have emerged as wide-spectrum, stress response factors protective in amyloid disease models. MDP cytoprotective functions are generally attributed to anti-apoptotic activity, however, little is known about their capacity to facilitate the cell’s unfolded protein response via direct interactions with amyloidogenic proteins. Here, we explored the effects of the MDP-analog, humaninS14G (HNG), and the MDP, small humanin-like peptide 2 (SHLP2), on the misfolding of islet amyloid polypeptide (IAPP), a critical pathogenic step in type 2 diabetes mellitus (T2DM). Our thioflavin T fluorescence studies show that HNG inhibits IAPP misfolding at highly substoichiometric concentrations. Seeded fluorescence and co-sedimentation studies demonstrate MDPs block amyloid seeding and directly bind misfolded, seeding-capable IAPP species. Furthermore, our electron paramagnetic resonance spectroscopy and circular dichroism data indicate MDPs do not act by binding IAPP monomers. Taken together our results reveal a novel chaperone-like activity wherein these MDPs specifically target misfolded amyloid seeds to inhibit IAPP misfolding which, along with direct anti-apoptotic activity and beneficial metabolic effects, make HNG and SHLP2 exciting prospects as T2DM therapeutics. These data also suggest that other mitochondrial stress response factors within the MDP family may be amenable to development into therapeutics for protein-misfolding diseases.

## Introduction

Mitochondrial-derived peptides (MDPs) are a family of polypeptides encoded in distinct open reading frames within the mitochondrial genome^1–3^. The 24 amino acid polypeptide, humanin (HN), was the first to be discovered and is the best-characterized among the identified MDPs^2^. Circulating levels of HN are associated with improved longevity in mouse models of aging^4^. This, combined with a diverse set of biological features ranging from cellular stress responses in multiple organ systems and tissues to modulation of metabolic activity^1, 5, 6^ have made humanin an attractive target for development as a therapeutic. Single amino acid substitutions have led to the discovery of humanin analogs with variable biological activity, such as the highly potent S14G-HN (HNG) humanin analogue^7–10^. In addition to humanin, other MDPs, including the small humanin-like peptide (SHLP) family and MOTS-c, have been recently reported to display similarly diverse biological features, by promoting cellular viability and reducing apoptosis in cell lines, as well as exhibiting beneficial insulin sensitizing effects *in vivo* ^2, 3, 11^. Thus, while humanin and its analogs are being investigated as potential therapeutics for degenerative, protein-misfolding diseases such as age-related macular degeneration (AMD), Alzheimer disease (AD), Creutzfeldt-Jakob disease (CJD) and diabetes mellitus (DM)^5, 6, 9, 12–15^, it appears that multiple MDPs may have therapeutic potential. Consistent with a role for MDPs as stress-inducible survival factors, the humanin homolog in rats, rattin, is upregulated in response to stressful stimuli^16^. In fact, humanin treatments rescue ROS-induced cytotoxicity in retinal pigmented epithelial models of AMD^12^, as well as amyloid-β (Aβ)^1, 17^ and prion-protein (PrP) oligomer-induced toxicity^14^, which are associated with AD and CJD, respectively. Many of the activities attributed to HN and HNG are thought to be mediated via signaling through cell surface receptors and interactions with intracellular apoptotic signaling molecules^18, 19^. Yet, given the spectrum of protein-misfolding diseases in which HN is protective, surprisingly little is known about whether humanin and other MDPs can directly influence the misfolding process via chaperone-like mechanisms.

Here we directly investigate this notion using the 37-amino acid polypeptide, islet amyloid polypeptide (IAPP), which plays a critical role in the pathogenesis of type 2 diabetes mellitus (T2DM)^20^. The misfolding and subsequent aggregation of IAPP induces a gain-of-function associated with β-cell apoptosis, oxidative damage, mitochondrial dysfunction and ER stress^21–24^. In the islets of Langerhans, the process of IAPP misfolding and aggregation ultimately leads to the replacement of pancreatic β-cell mass with deposits of fibrillar amyloid, which is the hallmark of T2DM pathology^25^. Within the cell, the formation of misfolded protein species puts demands on the cell for molecular chaperones to mitigate their deleterious effects, however, chaperone availability declines with age^26^, as does MDP availability^2, 6^ and with mounting stress, cellular proteostatic machinery can be overwhelmed leading to mitochondrial activation of apoptotic processes^26^. Given the general paucity of information available regarding MDPs and diabetes-associated protein-misfolding, plus observations that multiple MDPs display cytoprotective phenotypes^2, 15^, we sought to determine whether the potent humanin analog, HNG, as well as the MDP, SHLP2, could function in a chaperone-like capacity to prevent the misfolding of IAPP. We therefore used a combination of Thioflavin T (ThT) fluorescence studies in combination with electron paramagnetic resonance spectroscopy (EPR), circular dichroism (CD) and transmission electron microscopy (EM) to investigate the effect of HNG and SHLP2 on IAPP misfolding.

## Results

### MDPs inhibit IAPP misfolding

**Figure 1:**
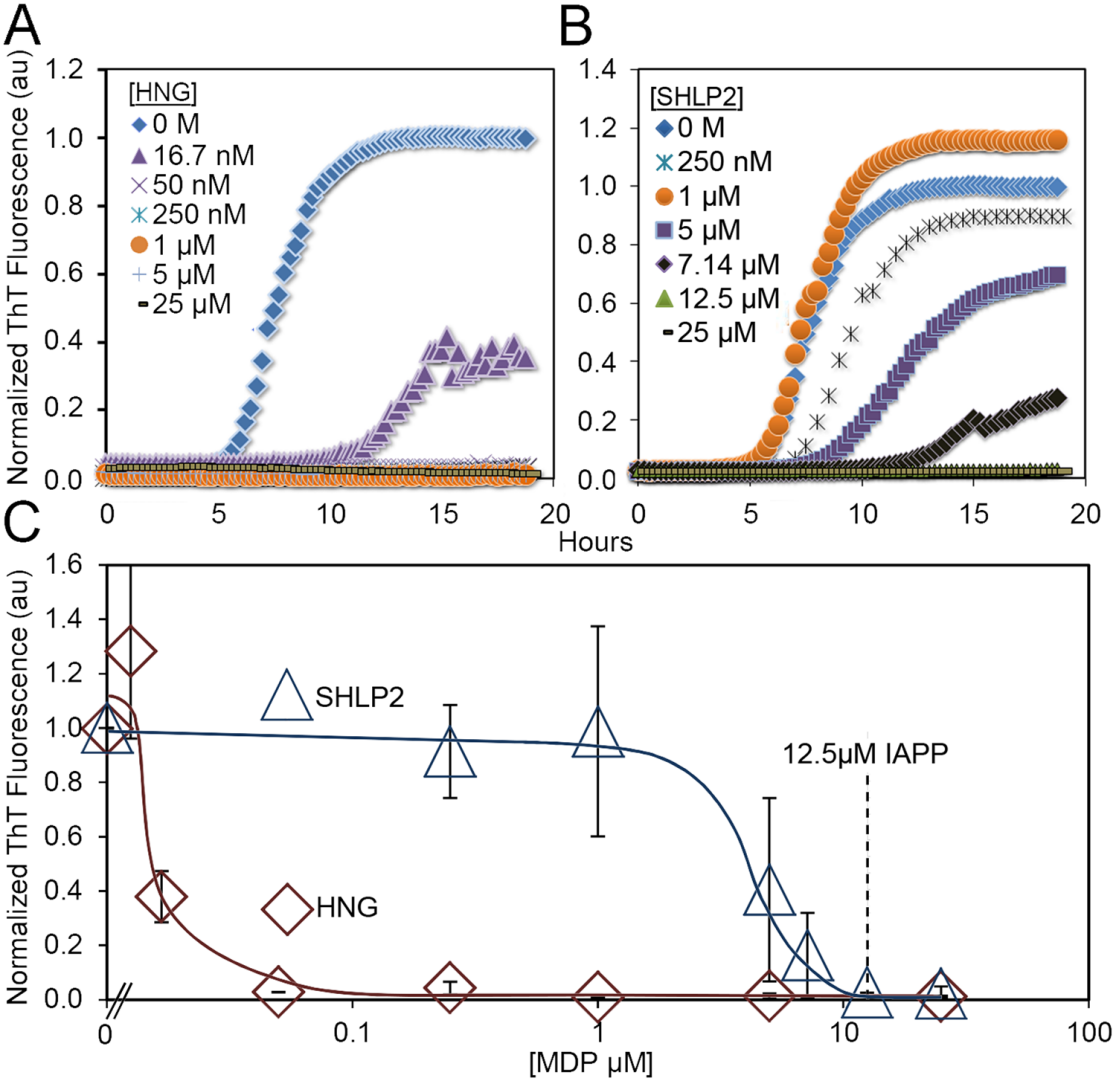
Mitochondrial-derived peptides HNG and SHLP2 inhibit IAPP fibrilization. (**A** and **B**) Representative ThT kinetics traces of IAPP misfolding in the presence of a) HNG or b) SHLP2. (**C**) End point analysis of IAPP misfolding by ThT fluorescence at 18 hours. Trend lines are shown for clarity. Concentrations of MDPs used to inhibit IAPP misfolding are given in micromolar below the figure. *p < *0.01*. Error bars represent +/− 1 standard deviation from a minimum of 3 experiments.

In order to determine whether mitochondrial-derived peptides have the capacity to inhibit the misfolding of islet amyloid polypeptide, we first chose two candidate MDPs to evaluate. We chose to test the HN analog HNG, where serine 14 is modified to a glycine residue, because of its significantly enhanced neuroprotective activity^7–10^. Small humanin-like peptide 2 (SHLP2) was chosen for study on account of its similar neuroprotective activity^2^. For the purposes of this paper, we will henceforth refer to both peptides as MDPs. We first monitored IAPP misfolding in the presence of HNG or SHLP2 using thioflavin T. For this experiment the IAPP concentration was held constant (at 12.5 μM) and the MDP concentrations were varied. To avoid complications from potential aggregation of HNG and SHLP2, we followed the peptide handling protocol first described by Arakawa *et al*.^27^ and only used fresh stocks of MDPs. According to our ThT data, both MDPs inhibit IAPP aggregation. We find that IAPP fibrilization, in the presence of either HNG or SHLP2, is reduced in a dose-dependent manner (Fig. 1A–C). In addition, kinetic analysis of IAPP misfolding demonstrates that both MDPs slow or entirely prevent the misfolding of IAPP within the timeframe of the experiment in a similarly dose-dependent manner (Supplemental Table 1a,b). HNG exhibited the greater potency, essentially completely inhibiting IAPP misfolding at substoichiometric concentrations, whereas closer to stoichiometric concentrations of SHLP2 were required. The remarkable ability of HNG to perform at substoichiometric concentrations (almost full inhibition at a molar ratio of 1:250, HNG:IAPP) implies that it is unlikely for HNG to act on the bulk of the monomeric IAPP to inhibit aggregation. Although binding to monomers could slow down aggregation by reducing the monomer pool available for misfolding, the strongly substoichiometric ratios would only allow a small subset of the IAPP molecules to be bound by HNG molecules. Such a minor reduction in available free IAPP would have negligible effects on the misfolding kinetics. SHLP2 also inhibits at sub-stoichiometric concentrations, however the effect is not nearly as pronounced as in the case of HNG. Therefore, we performed additional biophysical measurements to more clearly determine whether HNG, as well as SHLP2, act on species other than monomeric IAPP (see below).

### EPR Spectroscopy reveals MDPs prevent loss of monomeric IAPP without interactions with free IAPP

**Figure 2:**
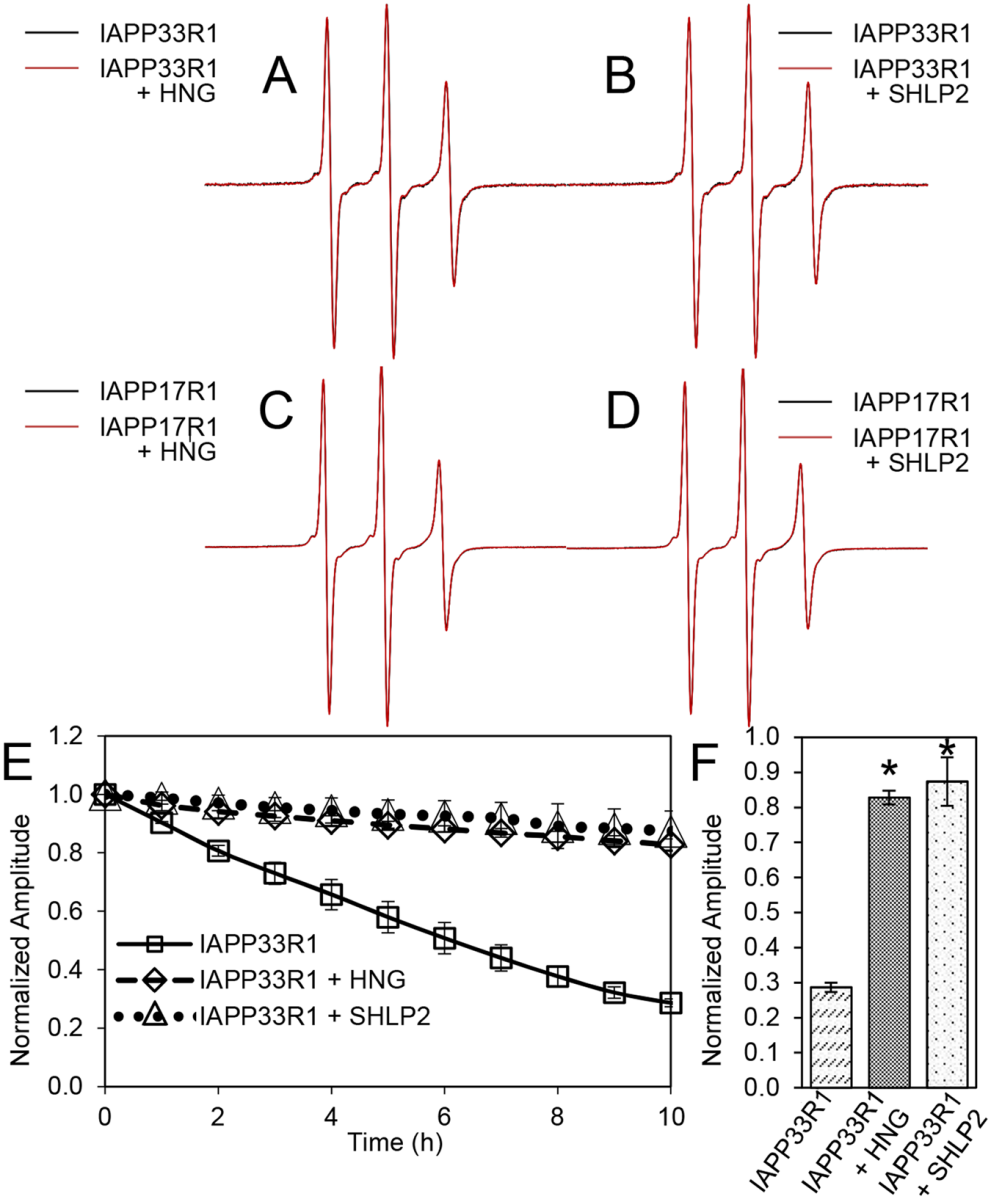
CW-EPR spectroscopy shows MDPs prevent loss of IAPP monomer without detectable binding to free IAPP. (**A**–**D**) EPR spectral overlay of (a) IAPP33R1 alone (black) and with HNG (red), (b) IAPP33R1 alone (black) and with SHLP2 (red), (c) IAPP17R1 alone (black) and with HNG (red), (d) IAPP17R1 alone (black) and with SHLP2 (red). (**E**) IAPP misfolding kinetics monitored over the course of fibrilization by central line amplitude. Symbols represent the following: (□) IAPP33R1, (◊) IAPP33R1 + HNG, (∆) IAPP33R1 + SHLP2. Error bars represent +/− 1 standard deviation. *p-values* of MDP treated vs. untreated IAPP33R1 at 1 hr <0.05, and at 2 + hr <0.01. (**F**) Average central line amplitudes for MDP treated IAPP33R1 at hour 10, *p = 0.001. Error bars represent +/− 1 standard deviation from a minimum of 3 experiments.

In order to better define the mechanisms by which MDPs inhibit IAPP misfolding, we used a combination of site-directed spin labelling (SDSL) and continuous wave-electron paramagnetic resonance spectroscopy. EPR spectra are highly sensitive to the mobility of the spin label and changes in their amplitudes can be used to monitor binding interactions and aggregation of free, monomeric IAPP^28–33^. Stoichiometric equivalents of MDPs were used for this experiment so that any MDP-IAPP(monomer) interactions would yield robust changes in central line amplitudes. Spectra were recorded for IAPP spin-labelled at amino acid 33 (IAPP33R1) and 17 (IAPP17R1)^30^ in the presence or absence of MDPs and their amplitudes were plotted (Fig. 2A–D). Spectra for both IAPP33R1 and IAPP17R1 showed sharp lines characteristic of a predominantly monomeric structure. When IAPP33R1 or IAPP17R1 were solubilized in the presence of HNG or SHLP2, we observed remarkably similar spectra for IAPP alone and in the presence of either MDP. Thus, no direct binding interactions between IAPP monomers and MDPs could be detected under the conditions used. Such binding interactions would have reduced the overall tumbling of IAPP and thereby yielded broader lines^32^. In addition, rigidification by binding or structuring within ~10 amino acids of the spin-labeled sidechains would have further resulted in line broadening^33^. These data are consistent with the sub-stoichiometric nature of the misfolding inhibition, further suggesting that HNG’s chaperone-like effect is likely not mediated by IAPP monomer binding. Inasmuch as we could not detect any line shape changes for SHLP2, it also appears that this functions via a mechanism other than monomer binding (also see below).

By following amplitude changes of IAPP33R1 EPR spectra over time, we can monitor misfolding in the presence and absence of MDPs as this region matures into a tightly packed, parallel in-register structure in fibrils^30^. When compared to untreated controls, both MDPs inhibited misfolding. In fact, we observed a remarkable retention of central line amplitude over the course of the experiment, with a slight but steady loss of amplitude in the case of both MDP treatments (Fig. 2E,F and Supplemental Figure S1). These data demonstrate that the presence of MDPs help the naïve population of IAPP largely retains its free, unaggregated state in solution. The slight changes in EPR signal indicate that, despite the presence of MDPs, small amounts of higher order IAPP species can still form.

### CD spectroscopy and TEM indicate MDPs prevent the misfolding of IAPP

**Figure 3:**
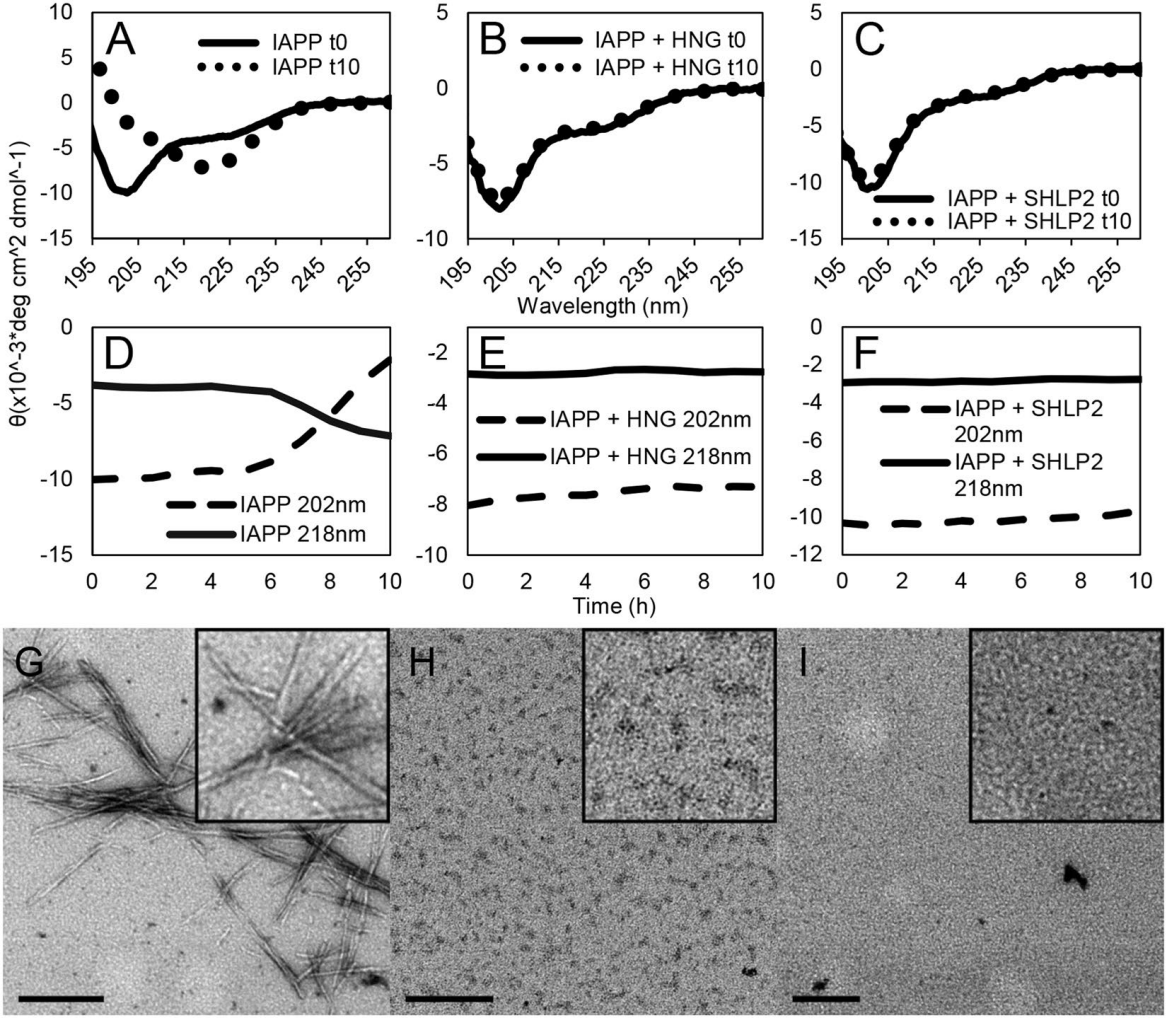
Time-resolved circular dichroism and transmission electron microscopy confirm MDPs prevent loss of IAPP monomers and prevent fibrilization. (**A**–**C**) Circular dichroism spectra of 15 μM IAPP with molar equivalents of MDPs recorded at the beginning and end of each experiment, displayed as a weighted mean residual ellipticity (MRE’). For details regarding the calculation of MRE‘ see Materials and Methods section. (**D**–**F**) Time resolved CD of experiments in (a–c). Ellipticities were recorded at 202 nm and 218 nm to follow transitions from random coil (202 nm) to β-sheet (218 nm). Traces represent an average of at least 3 experiments. (**G**–**I**) Electron micrographs taken at the end of the experiment for (**G**) IAPP alone, (**H**) IAPP treated with HNG, and (**I**) IAPP treated with SHLP2. Scale bars equal 200 nm.

In order to monitor the secondary structure changes during IAPP misfolding in the presence or absence of MDPs, we performed time-resolved circular dichroism. For these experiments we mimicked the conditions used in the EPR experiments, that is, 10 mM phosphate buffer, pH 7.4 with stoichiometric equivalents of IAPP and HNG or SHLP2. We first monitored secondary structural changes of IAPP and MDPs taken in isolation. As expected, IAPP alone in solution is initially largely disordered as indicated by a negative peak at ~202 nm (Fig. 3A,D). Over the course of fibrilization, IAPP develops a negative peak at 218 nm, indicating the formation of β-sheet rich conformers. Electron microscopy confirms the development of IAPP fibrils (Fig. 3G). On the other hand, HNG and SHLP2 in the same conditions retain a primarily random coil signal throughout the duration of the experiment (Supplemental Figure S2a–d).

We then used CD to monitor IAPP misfolding in the presence of MDPs. For early time points, we find that the spectra of the individual peptides add, arithmetically, to match those of the peptides together (Figure S3a,b). These data agree with our EPR findings showing no significant interactions between the free IAPP population and either MDP.

We next used CD to follow IAPP treated with MDPs over time. Whether treated with HNG or SHLP2, the IAPP MDP mixtures mostly retained their starting secondary structures, predominantly random coil (Fig. 3B,C,E,F). The observation of largely monomeric samples devoid of significant aggregates is consistent with EM images of MDP-treated IAPP (Fig. 3H,I). In contrast to control samples from IAPP alone, fibrils are largely absent in the presence of MDPs. Instead, we see a few small structures approaching the limit of our resolution in MDP-treated samples of IAPP similar to those seen when the MDPs are observed by EM alone (Supplemental Figure S4) that may represent multimeric MDP complexes or IAPP:MDP complexes. The CD and EM data are in direct agreement with our EPR studies and validate the notion that MDPs maintain the bulk of IAPP in a monomeric state.

Reminiscent of our EPR observations, there is a small loss of random coil signal over time in IAPP samples treated with either HNG or SHLP2 (Fig. 3E,F, dashed lines). While the CD data do not indicate which of the peptides is responsible for this slow conformational change, our EPR data indicate that structural changes in the IAPP must have at least in part been responsible for the observed changes seen by CD.

### HNG and SHLP2 prevent propagation by IAPP seeds

**Figure 4:**
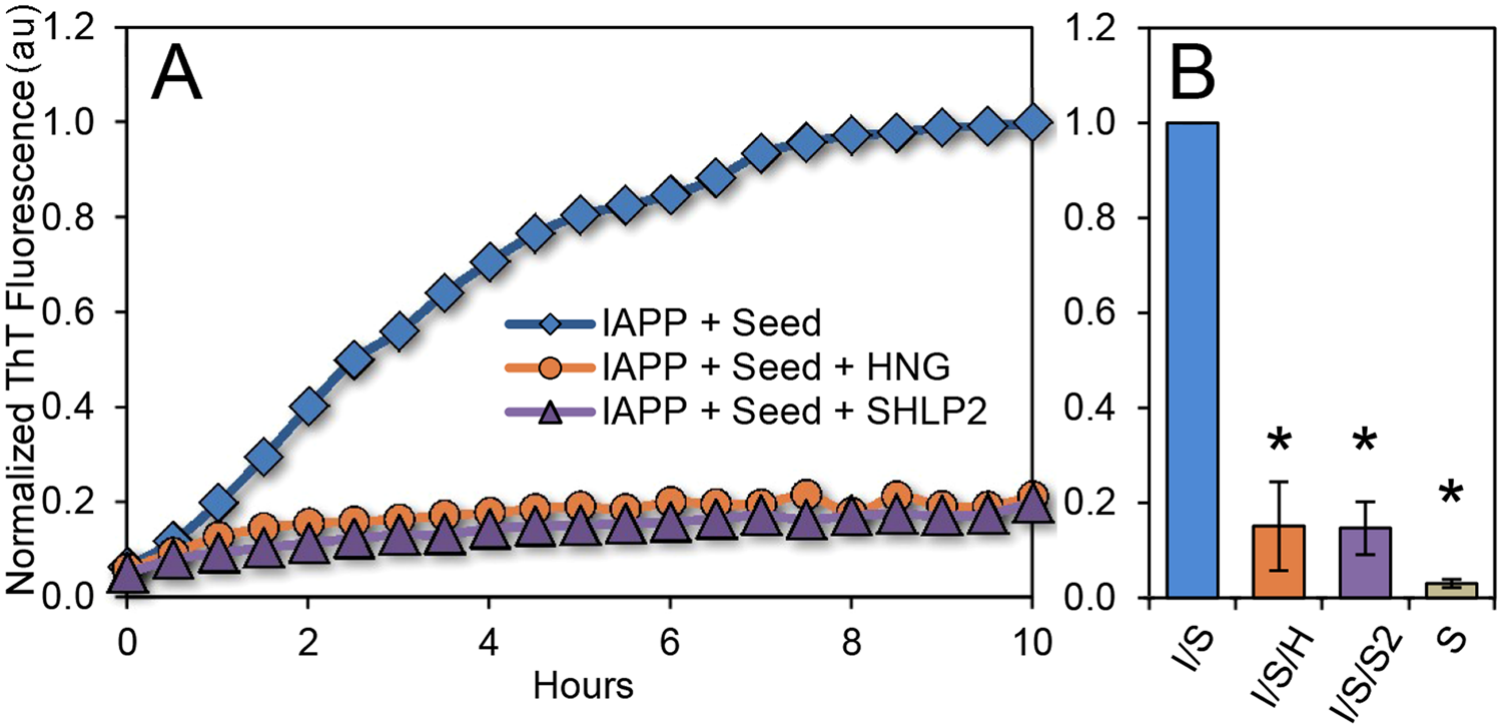
MDPs protect against seeding of pre-formed IAPP aggregates. (**A**) Representative fluorescence kinetics traces from 2.5 mol % seeded 12.5 μM IAPP reactions treated with HNG or SHLP2. (**B**) Bar graph of averaged and normalized fluorescence data from (a) at 10 hours. Error bars represent +/− standard deviation from at least 3 independent measurements. *p < 0.01.

The ThT, EPR and CD are inconsistent with a mechanism in which the MDPs affect misfolding via binding to IAPP monomers. An alternative way in which MDPs could prevent misfolding without interacting with the monomer is to prevent seeds from promoting misfolding. In order to test this, we sonicated pre-formed IAPP fibrils and used them to seed freshly dissolved IAPP. Once again we monitored the misfolding of IAPP in the presence or absence of HNG or SHLP2 using ThT. As is expected from a seeded reaction, we observed that seeding bypasses the lag phase in the absence of MDPs. Furthermore, we found that both HNG and SHLP2 displayed remarkable capacity to inhibit the seeded IAPP reaction (Fig. 4A,B). This, in concert with our earlier ThT, EPR and CD data implies that MDPs bind directly to misfolded IAPP seeds.

### MDPs directly interact with IAPP seeds

**Figure 5:**
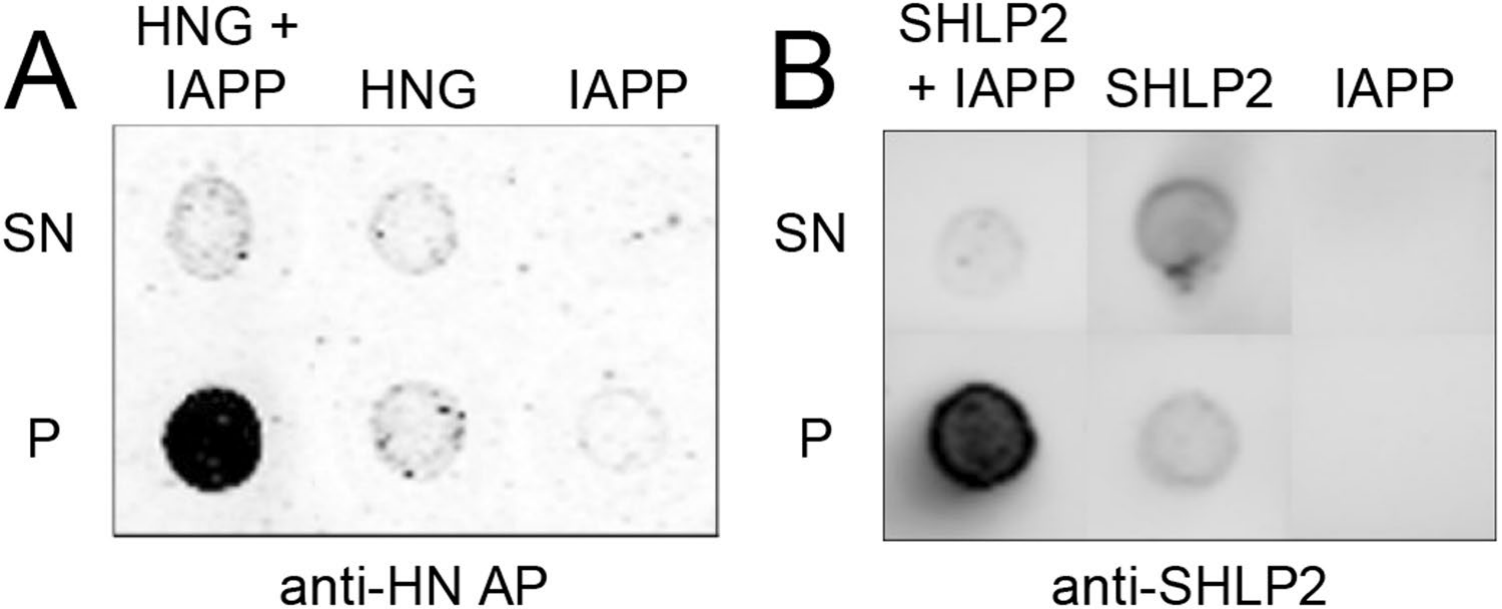
Co-sedimentation assay shows HNG and SHLP2 bind IAPP seeds. (**A**) Representative dot blot from supernatant (top) and pelleted (bottom) fractions of HNG and sonicated pre-formed IAPP fibrils (HNG + IAPP), HNG alone (HNG), or sonicated pre-formed IAPP fibrils alone (IAPP), probed with α-HN AP antibody. (**B**) Representative dot blot from supernatant (top) and pelleted (bottom) fractions of SHLP2 and sonicated pre-formed IAPP fibrils (SHLP2 + IAPP), SHLP2 alone (SHLP2), or sonicated pre-formed IAPP fibrils alone (IAPP), probed with α-SHLP2 antibody.

In order to test for a direct interaction between the MDPs and IAPP seeds, we employed a co-sedimentation assay. Sonicated seeds, despite their reduced size, can be pelleted through ultracentrifugation, allowing us to enrich pellets with IAPP seeds and anything that they pull down with them. We, therefore, incubated sonicated pre-formed IAPP fibrils with HNG or SHLP2 and pelleted the seed fraction. Membranes dotted with supernatant and pellet samples were probed with antibodies against either HNG (anti-HN AP) or SHLP2 (anti-SHLP2). These experiments were performed in relatively dilute conditions, near the detection limit of the antibodies, however, in both cases, we found that the pelleted fraction was highly enriched in MDPs (Fig. 5), verifying that HNG and SHLP2 directly interact with IAPP seeds. This finding is consistent with a mechanism wherein MDPs inhibit IAPP misfolding by preferentially targeting misfolded IAPP. As small volumes of supernatant were allowed to remain in the pellet fraction to prevent accidental removal of the pellet, it is expected that trace amounts of soluble MDP are observed in the pellet fractions from samples containing only HNG or SHLP2.

## Discussion

Here we show that the mitochondrial-derived peptides HNG and SHLP2 can prevent the amyloid formation of IAPP. Multiple lines of evidence indicate that the mechanism underlying this activity is the binding of misfolded IAPP seeds by the two MDPs. The strongly substoichiometric inhibition of IAPP misfolding by HNG rules out the necessity for bulk capture of monomeric IAPP by HNG. Moreover, the EPR data do not detect any binding interaction between free monomeric IAPP and either MDP, while the CD data reveal that mixing of IAPP and MDPs does not result in detectable changes in secondary structure for either of the peptides. These data are inconsistent with any substantive binding of the free pool of naïve bulk IAPP by either MDP. Binding to a small subset of seeding competent monomers could, at least in principle be possible. However, there is no evidence that such monomers exist for IAPP and it is well-known that multimeric species, like the ones used here, can be potent seeds. In fact, we observe direct interactions between the MDPs and multimeric IAPP misfolding seeds by co-sedimentation. This interaction is sufficient to robustly prevent seeded misfolding reactions indicating that these MDPs have the capacity to prevent such seeds from functioning as a template for the misfolding of naïve IAPP. Consistent with this notion, our CD and EPR data further demonstrate that the hallmark cooperativity normally observed in IAPP amyloid formation reactions can be blocked by MDP treatments. In kinetics terms, we observe effects by both MDPs on the lag phase of the ThT tracings, as well as the steepness of the slope once fibrilization is underway at higher inhibitor concentrations. At the lowest inhibitor concentrations that don’t significantly extend *t*_50_ values, we do not observe slope changes. This combination of kinetics findings are mechanistically explainable by the prevention of propagation of the amyloid template by the MDPs, but does not rule out inhibitory effects on primary nucleation^34^.

A subtle feature observed in the inhibition of IAPP misfolding by MDPs is the slow decrease in free IAPP signal observed in both CD and EPR studies. Combined with the lack of cooperativity in the process of oligomerization, these data suggest that some higher order species can, in fact, form in the presence of MDPs but that they cannot efficiently promote seeding. In light of these findings, it seems unlikely that MDPs act by completely blocking the formation of all seeding capable oligomeric and fibrillar species, as has been seen in the case of some chaperones^34, 35^. Rather, these data are consistent with a mechanism wherein MDPs bind to any seeds that may have formed, and, in doing so, prevent the propagation of their fold. As such, this effect is mechanistically akin to the “cap and contain” mechanism previously described for the chaperonins TRiC and CCT5, which bind to misfolded huntingtin in a manner that blocks seeding-mediated misfolding from both oligomeric and fibrillar species^36^. Future work will have to reveal whether such a mechanism indeed applies to MDPs.

The chaperone-like function of the MDPs observed here also provides an additional likely mechanistic explanation to findings from studies showing that HN protects in the setting of Aβ toxicity and AD pathology. Indeed, it was recently found that HN and HNG interact with Aβ oligomers^17^. We speculate that such chaperone-like activity, when taken in context with the known neuroprotective effects of HN, raises the possibility that HN and other MDPs serve a role as a sensor of, or a response to, aberrant protein or amyloid misfolding. If so, this would expand the function of mitochondria in amyloid disease beyond its apoptotic role to that of an early component of the cellular defense system.

Previous studies have shown that HN has the ability to self-assemble^37^ and that this ability correlates with higher cytoprotective activity^7, 8, 38^. One of the open questions is whether the amyloid inhibitory effect of HNG and SHLP2 is mediated by their monomers or oligomers. Our EM data indicate that oligomers form even in the absence of IAPP. Such oligomers could act in a manner similar to the cap and contain mechanism observed for chaperone-like proteins. We noticed in the present study that freshly prepared MDPs have a higher activity. Thus, if oligomers represent the active species, then they are likely to be early and potentially smaller oligomers.

The cytoprotective functions of HN and its analogs are well-described, yet, much remains to be determined regarding the mechanisms by which MDPs protect cells from various insults. HN and its derivatives (e.g. HNG) are known to function via at least three distinct biological mechanisms, including activation of the STAT3 pathway, ERK1/2 pathway, and via interactions with apoptotic regulators Bax and IGFBP-3^18^. Here we have shown that MDPs can directly modulate amyloid misfolding via a chaperone-like mechanism. Dissecting the relative cellular and organismal levels contributions of these functions and the chaperone-like effects we have described herein will be a complex but informative endeavor.

The data presented here also highlight the therapeutic potential of HNG, SHLP2 or related molecules in the treatment of T2DM. Our data reveal that interactions of MDPs with IAPP have a marked degree of specificity for non-monomeric IAPP species. This quality has two beneficial aspects. First, it is likely to reduce interactions that prevent IAPP monomers from performing their regular physiological function, and second, by inhibiting seeding only a small amount of misfolding inhibitor is needed. These are attractive drug properties as they will likely help to reduce dosage and potential side effects. Taken in context with the pleiotropic qualities already assigned to humanin, these properties make therapeutic approaches with humanin, SHLP2 and perhaps other MDPs an exciting avenue for further research. Such research may also advance our understanding of how to target seeds rather than monomers and allow for the development of molecules with similar functional properties. Finally, it will be important to investigate the degree to which all the members of the MDP family have similar chaperone-like qualities to protect against various forms of protein misfolding and which oligomerization state mediates their chaperone-like activities.

## Experimental procedures

### Materials and chemicals

Wild-type human IAPP was purchased from Bachem America (Torrance, CA). Hexafluoroisopropanol was purchased from Sigma-Aldrich. 1-oxyl-2,2,5,5-tetramethyl-Δ3-pyrroline-3-methyl methanethiosulfonate (MTSL), was purchased from Toronto Research Chemicals (Toronto, Ontario, Canada). Human IAPP cysteine mutants with alanine substitutions for the native cysteines at positions 2 and 7 were purchased from Biomer Technology (Pleasanton, CA). Humanin S14G (HNG) was obtained from Genscript (Piscataway, NJ). Small humanin-like peptide 2 (SHLP2) was obtained from CPC Scientific (San Jose, CA). SuperBlock T20(PBS) Blocking Buffer was obtained from Thermo Scientific (Rockford, IL). Antibodies against humanin (anti-HN AP) and SHLP2 (anti-SHLP2) were generated by YenZym (San Francisco, CA) and Harlan (Indianapolis, IN), respectively.

### Peptide handling and storage

Lyophilized wild type human IAPP was dissolved in HFIP, aliquoted into individual tubes and flash frozen in N_2_ (*l*) prior to lyophilization. UV absorbance at 280 nm was used to determine IAPP protein concentrations in denaturing conditions (8 M guanidinium chloride) using an ε_280_ of 1405 M^−1^cm^−1^ and verified by CD spectroscopy upon resolublization. Lyophilized IAPP stocks were stored in N_2_ (g) under vacuum. MTSL labeled IAPP was stored at −20 °C in HFIP. HNG was obtained lyophilized from Genscript and stored lyophilized at −80 °C until solubilization. Solubilization and storage of HNG was performed according to the method described by Arakawa *et al*.^27^. Briefly, HNG was solubilized in water at 1 mg/mL and aliquoted. Aliquots were stored at −20 °C until use. SHLP2 was obtained lyophilized and stored at −80 °C until used. SHLP2 was solubilized in water at 1 mg/mL, aliquoted and stored at −20 °C until use. We observed that prolonged storage (>1 month) of solubilized HNG stocks at −20 °C markedly attenuated the potency of HNG and special care was taken to work with fresh stocks of HNG. With SHLP2 this effect was less pronounced, but similar care was nevertheless taken to work with fresh stocks of SHLP2.

### Peptide labeling

Spin labelling was performed as before^30^. Briefly, single cysteine mutants of IAPP were incubated with MTSL (>5 molar excess) for ~1 h at room temperature. Excess MTSL was removed via cation exchange using a Toyopearl cation exchange column and subsequently desalted on a C18 reverse phase SpinColumn (Harvard Apparatus, Holliston, MA), and ultimately eluted in HFIP. Spin labelled peptide was stored in HFIP at −20 °C. Peptide concentration was verified at the beginning of each experiment by comparing central line amplitudes and double integral values against a standard concentration curve on the EPR apparatus.

### Thioflavin T Fluorescence Studies

Thioflavin T (ThT) was stored at a 5 mM stock concentration in water at −20 °C. ThT was used at a 25 μM final concentration to monitor IAPP misfolding. IAPP aliquots were prepared as above. Individual samples of IAPP were solubilized in appropriate buffer with ThT to a concentration of 12.5 μM from a dry powder in 10 mM potassium phosphate buffer, pH 7.4. MDPs were prepared to a stock concentration of 1 mg/mL as described above and added to appropriate reactions. The mixtures were monitored for fluorescence in a 2 mm quartz cuvette and a Jasco FP-6500 spectrofluorometer at room temperature. Fluorescence was monitored under the following settings and conditions: excitation wavelength = 450 nm, emission wavelength = 482 nm, excitation slit width = 1 nm, emission slit width = 10 nm, and pH = 7.4. *t*_50_ values were determined as before^39, 40^, using a sigmoidal model to fit our data. Each experiment was normalized to an appropriate IAPP control by dividing fluorescence intensities by the maximal IAPP control intensity. Statistical analysis for comparison was performed using the student t-test.

For seeding experiments IAPP was fibrilized in 10 mM phosphate buffer, pH 7.4 for 2 weeks at 55 μM. Fibrils were sonicated using a titanium tip sonicator 4 × 30 seconds each and placed on ice in between sonications. ThT, IAPP and MDPs were prepared as described above. Fluorescence was measured in an Eppendorf AF2200 96-well fluorescence plate reader. Reaction volumes were ~100 μL. Fluorescence was monitored under the following settings and conditions: excitation wavelength = 440 nm, emission wavelength = 484 nm, 25 flashes per measurement and a gain setting of 75.

### Co-sedimentation Assay

For determination of direct interaction between HNG and IAPP, 4:1 wt/wt ratios of HNG and sonicated pre-formed IAPP fibrils (1 µg: 250 ng, respectively), or 1:1 wt/wt ratios of SHLP2 and sonicated pre-formed fibrils (1 µg:1 µg, respectively) were co-incubated in a 50 µL reaction volume and centrifuged at 55,000 rpm for 30 minutes at 4 °C. 90% of the reaction volumes were removed and labeled as supernatant. 20 µL of buffer was added to the pellet fraction to facilitate efficient recovery. Buffer was added to supernatant fractions to appropriately match pellet dilution and 2 µL dots from supernatant and pellet samples were placed on nitrocellulose membranes then dried. Membranes were subsequently blocked for 30–60 minutes in either milk or SuperBlock T20(PBS) Blocking Buffer and probed with antibody against either HNG (1:1000, α-HN AP; chicken) or SHLP2 (1:500, α-SHLP2; rabbit)^2^ for 1 hour at room temperature or overnight at 4 °C. Primary antibody was recovered and membranes were washed 3 × 5 min in tris buffered saline with 0.005% tween 20 (TBST). HNG and SHLP2 treated membranes were then probed with IRdye 800 donkey α-chicken (1:10,000 Li-Cor) and IRdye 680 donkey α-rabbit (1:10,000 Li-Cor). Membranes were washed 3 × 15 minutes in TBST and imaged on a Li-Cor Odyssey fluorescent imager.

### Circular Dichroism Spectroscopy

IAPP was prepared as above. CD spectra were measured between 195 and 260 nm in a Jasco-815 spectropolarimeter. Spectra were scanned at a rate of 50 nm/min and measurements taken every 0.5 nm, with an averaging time of 1 second and backgrounds were subtracted with measurements made from buffer alone (i.e. 10 mM phosphate buffer, pH 7.4). Time-resolved measurements were taken at 202 or 218 nm and averaged over 20 seconds. Ellipticity values were converted into mean residual ellipticity using equation (1)

1 $$\theta =\frac{\theta raw}{nCl}\,,$$

where *θ* is the mean residual ellipticity expressed in degrees cm^2^ dmol^−1^, *θ*_*raw*_ is the measured ellipticity in millidegrees, *n* is the number of amino acids, *C* is the molar concentration, and *l* is the path length in millimeters.

### Mean residual ellipticity calculations in mixed CD samples (MRE‘)

For CD experiments where IAPP and either HNG or SHLP2 were measured together, MRE‘ values are sometimes reported. We calculated MRE‘ values by converting equation [1] to equation [2]

2 $$\theta =\frac{{\theta }_{raw}}{(({n}_{1}\times {C}_{1})+({n}_{2}\times {C}_{2}))l},$$

Where n_1_ and C_1_ represent the number of amino acids and concentration of one peptide, respectively, and n_2_ and C_2_ represent the number of amino acids and concentration of the second peptide.

For initial interaction studies, addition of IAPP and MDP alone values were performed using JASCO Spectra Manager 2.0 software. In this case, ellipticities are reported in their raw form.

### Electron Paramagnetic Resonance Spectroscopy

Cys-less IAPP33C was labeled with MTSL (IAPP33R1) and stored as described above. Stock IAPP33R1 was dried in a constant stream of N_2_ (g) and solubilized to a concentration of ~15 μM in 10 mM phosphate buffer pH 7.4 with either vehicle or stoichiometric equivalents of MDP. Samples were drawn up into glass capillaries (0.6 mm diameter, VitroCom, Mountain Lakes, NJ) and sealed at the end. Spectra were recorded on a Bruker EMX spectrometer (Billerica, MA) at 12 mW with a magnetic field scan range of 100 Gauss in an HS resonator. Measurements of central line amplitude were made by taking the difference between the amplitudes (in arbitrary units) of the zenith and nadir of the central line of each EPR spectrum, as previously published^28, 41^. Values were normalized to the starting amplitude and statistical p-values determined by student t-test.

### Transmission Electron Microscopy

Samples were applied to carbon-coated Formvar films mounted on copper grids for at least 10 minutes and negative stained with 1% uranyl acetate after excess liquid was blotted away. Grids were imaged on a Jeol-1400 transmission electron microscope operated at 100 kV.

All data generated or analyzed during this study are included in this published article (and its Supplementary Information files).

## Electronic supplementary material

Supplementary Info
