# Supplementary material for: The Mitochondrial-Derived Peptides, HumaninS14G and Small Humanin-like Peptide 2, Exhibit Chaperone-like Activity
Source: Sci Rep. 2017 Aug 10;7:7802. doi: 10.1038/s41598-017-08372-5 (PMC5552803; doi:10.1038/s41598-017-08372-5)
Supplement: Supplementary file 1 — Supplementary Info [file 41598_2017_8372_MOESM1_ESM.pdf]

The Mitochondrial-Derived Peptides, HumaninS14G and Small Humanin-like Peptide 2, Exhibit Chaperone-like Activity

Alan K. Okada[1*], Kazuki Teranishi[1*], Fleur Lobo[1], J. Mario Isas[1], Jialin Xiao[2], Kelvin Yen[2], Pinchas Cohen[2], Ralf Langen[1¥]

[1]Department of Biochemistry and Molecular Biology, Zilkha Neurogenetic Institute, University of Southern California, Los Angeles, California 90033, USA, [2]University of Southern California Davis School of Gerontology, Ethel Percy Andrus Gerontology Center, University of Southern California, Los Angeles, CA 90089-0191, USA

[¥]Corresponding Author: Ralf Langen, Zilkha Neurogenetic Institute, Department of Biochemistry and Molecular Biology, University of Southern California, Los Angeles, California 90033, USA. Tel.: 323-442-1323 Fax: 323-442-4404. Email: Langen@usc.edu
* These authors contributed equally to this work

Table S1

A

HNG (μM)	12.5 μM IAPP t_{50} (h)
0	8.4 ± 1.3
0.0125	13.4 ± 0.5
0.0167	12.4 ± 2.6
0.05	>20
0.25	>20
1	>20
5	>20
25	>20

B

SHLP2 (μM)	12.5 μM IAPP t_{50} (h)
0	8.4 ± 1.3
0.25	10.2 ± 2.7
1	11.1 ± 3.0
5	13.1 ± 2.3
7.14	>20
12.5	>20
25	>20

Table S1 – *MDPs HNG and SHLP2 inhibit the misfolding of IAPP*. A and B) Kinetics study of IAPP misfolding by ThT fluorescence in the presence of varying concentrations of (a) HNG or (b) SHLP2. Mean t_{50} values from the experiments performed in Figure 1 are shown in the table. MDP concentrations are given in μM starting at 0, in the absence of MDPs, and t_{50} averages are given in hours. Data are presented as an average t_{50} ± 1 standard deviation. For conditions where no fibrilization was observed over the course of the experiment, a >20h is given. Refer to Figure 1 for methods.

Figure S1

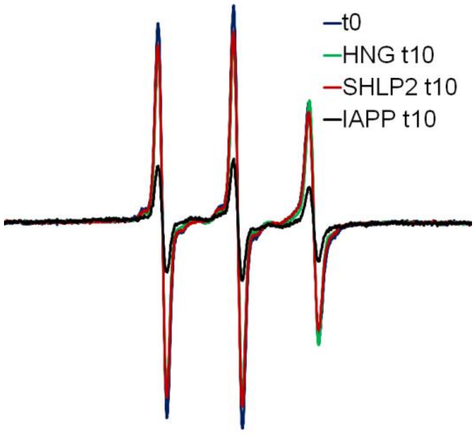

Figure S1. *CW-EPR spectroscopy shows that MDPs largely prevent the cooperative aggregation of IAPP*. Overlay of EPR spectra taken of IAPP33R1 after 10 hours alone (black), in the presence of SHLP2 (red), or HNG (green) are shown as in Figure 2. The spectra recorded at time zero (t0 - blue) is included for reference. Refer to Figure 2 for methods.

Figure S2

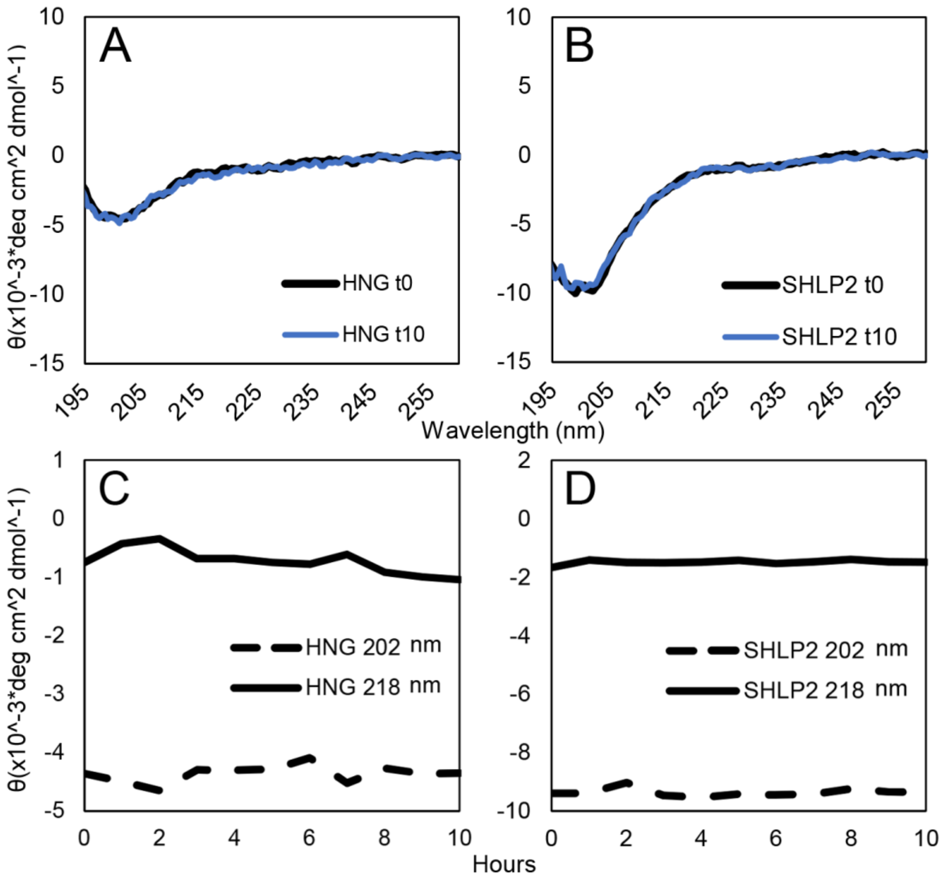

Figure S2. *HNG and SHLP2 structural stability during time-resolved circular dichroism*. A) Spectra of HNG taken at the beginning and end of time-resolved CD experiments. B) Spectra of SHLP2 at the beginning and end of time-resolved CD experiments. C) Time-resolved ellipticity of HNG recorded at 202 and 218 nm. D) Time-resolved ellipticity of SHLP2 recorded at 202 and 218 nm. Traces are an average of at least 3 experiments.

Figure S3

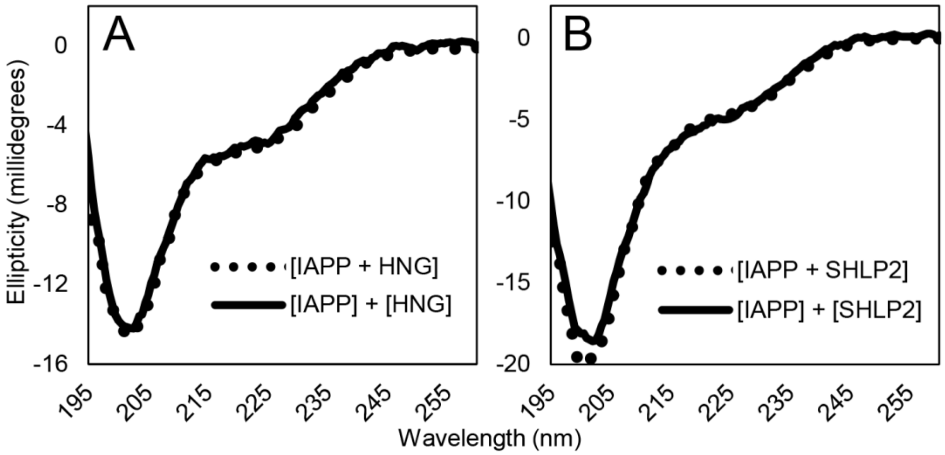

Figure S3. *IAPP and MDPs alone and together remain primarily disordered in solution*. A) CD spectra of the arithmetic sum of IAPP and HNG measured alone, [IAPP] + [HNG] (solid line), overlaid with the spectra of IAPP and HNG mixed in solution, [IAPP + HNG] (dotted line). B) Spectra of IAPP and SHLP2 measured separately, [IAPP] + [SHLP2] (solid line), overlaid with IAPP and SHLP2 together in solution, [IAPP + SHLP2] (dotted line). Traces are an average of at least 3 experiments.

Figure S4

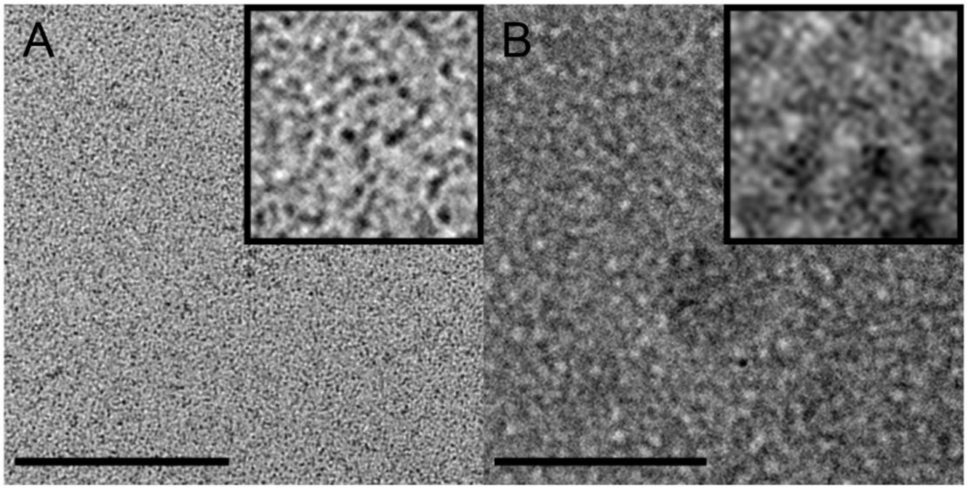

Figure S4. *MDPs HNG and SHLP2 form small opaque structures by EM*. A and B) Electron micrographs of 15 µM MDPs incubated without IAPP for 10 hours are shown for A) HNG alone and B) SHLP2 alone. Scale bars equal 200 nm.
